# Melanoma Arising after Imiquimod Use

**DOI:** 10.1155/2014/267535

**Published:** 2014-11-09

**Authors:** Sharad P. Paul

**Affiliations:** ^1^University of Auckland, Auckland 1010, New Zealand; ^2^University of Queensland, Brisbane, QLD 4072, Australia; ^3^Skin Surgery Clinic, Auckland 0600, New Zealand

## Abstract

Imiquimod belongs to the class of 1H-imidazo-[4,5-c]quinolones—drugs originally developed as nucleoside analogues with the aim of finding new potential antiviral agents (Harrison et al., 1988). Indeed, Imiquimod was first released as treatment for genital warts before its actions against skin cancer were studied. Imiquimod is a relatively small sized molecule (Mr = 240.3) and is hydrophobic, allowing it to penetrate the skin epidermal barrier and therefore making it suitable for topical formulations (Gerster et al., 2005). Imiquimod has shown itself effective against skin cancers and precancerous lesions, especially basal cell cancers and actinic keratosis (Salasche et al., 2002, Beutner et al., 1999). There have been reports of Imiquimod being used as topical treatment against cutaneous metastases of melanoma and some authors have reported its use as first-line therapy against melanoma in situ (Smyth et al., 2011, Gagnon, 2011). We report a case of an invasive malignant melanoma arising de novo at the specific site of application of Imiquimod (Aldara cream 5%) for a biopsy-proven superficial BCC. Therefore while Imiquimod has added to our topical armamentarium against skin cancer, care must be exercised in prescribing this treatment and it is especially important to follow up patients regularly.

## 1. Background

Imiquimod belongs to the class of 1H-imidazo-[4,5-c]quinolones—a group of drugs that was originally developed as nucleoside analogues with the aim to find new potential antiviral agents [1]. Indeed, Imiquimod was first released as treatment for genital warts before its actions against skin cancer were studied. Imiquimod is a relatively small sized molecule (Mr = 240.3). The molecular size, as well as it being hydrophobic, allow it to penetrate the skin epidermal barrier and therefore make it suitable for topical formulations [2]. In many studies Imiquimod has shown itself effective against skin cancers and pre-cancerous lesions, especially basal cell cancers and actinic keratosis [3, 4]. There have also been reports of Imiquimod being used as topical treatment against cutaneous metastases of melanoma and some authors have reported its use as first-line therapy against melanoma in situ [5, 6].

In recent years, Imiquimod has become widely used as topical treatment for skin cancers. Its tumouricidal activity is based mainly on activating the innate immune system, for which dendritic cells seem primarily responsible. These dendritic cells initiate a tumour-directed cellular immune response [7]. Researchers have noted that dendritic cells respond to much lower concentrations of Imiquimod than many other cell types [8]. At higher, but therapeutically relevant, concentrations, Imiquimod exerts some proapoptotic activity against tumour cells.

Toll-like receptors (TLRs), especially TLR 7 and TLR 8, are important receptors of this innate immune system. It is generally felt that Imiquimod is an agonist of TLRs 7 and 8 [9]. However, while these innate immunity-related actions are well known, there are some findings which cannot be explained easily by TLR-dependent mechanisms—for example, Imidazoquinolines like Imiquimod can stimulate the proliferation of B cells in vitro, even in the absence of other immunocytes [10].

However, in recent times Imiquimod has been shown to paradoxically cause tumours, or more precisely tumours have been reported at bodily sites of treatment. In 2006, two cases of invasive SCC arising after treatment of squamous carcinoma in situ with 5% Imiquimod cream were reported [11]. While the exact mechanism of tumour-induction by Imiquimod is unclear, presumably it is due to its local alteration and stimulation of an exuberant immune response. Keratoacanthomas have also been reported as arising after treatment with topical Imiquimod [12].

Some authors have used Imiquimod “off-label” and have reported resolution of primary melanoma in situ (lentigo maligna) and recurrent lentigo maligna with 5% Imiquimod cream [13, 14]. Some authors have also noted Imiquimod inhibits melanoma development by promoting pDC cytotoxic functions and impeding tumour vascularization [15], and there have been many reports where researchers have used Imiquimod topically to treat melanoma metastases [16].

In this context, we believe our case report to be noteworthy and worth reporting as, in our patient, 5% Imiquimod was used as topical treatment for a biopsy-proven BCC and the patient ended up developing an invasive melanoma over the site, while, as discussed earlier, keratoacanthomas have been known to develop at the precise site of a treated superficial BCC—an invasive melanoma arising in this situation is unusual and, to our knowledge, has not been reported previously. In the case of our patient, the area on his back was marked for treatment, which was then undertaken for 6 weeks with 5% Imiquimod (Aldara cream) with two treatment-free days each week as per usual protocol. At 8 weeks, when the patient was reviewed, he had a complete clearance of the BCC noted earlier; however, he had developed a new pigmented lesion over the site of topical application of Imiquimod which both on dermoscopy and on clinical examination was suspicious for melanoma. Histopathological analysis has confirmed this to be an invasive melanoma. While many authors are advocating the use of Imiquimod for melanoma, we would like to present this case, where an invasive melanoma has arisen at the precise site of application of Imiquimod (Aldara cream 5%) for a superficial BCC.

## 2. Case History

A 60-year-old white male presented to our skin cancer centre with superficial BCC areas on his mid back. Given that he had three to four sBCCs present within a 10 cm area, it was decided to treat these lesions topically using Imiquimod (Aldara cream 5%). A biopsy was undertaken initially to confirm sBCC. We used the standard protocol recommended by the manufacturers; that is, the cream was applied to the affected area once a day at bedtime for five consecutive days per week (Monday to Friday) for 6 weeks. The patient was reviewed at 8 weeks and it was noted that the patient had developed a de novo pigmented lesion over the site of application of Imiquimod. Given that the clinical impression was that of a malignant melanoma, this lesion was excised.

### 2.1. Histopathology

*Specimen*

 Excision skin lesion back.

*Gross Description*

 The specimen consisting of a skin ellipse 15 mm × 10 mm × 5 mm bearing a central dark brown irregular lesion approximately 9 mm × 7 mm. 3r 6l.

*Microscopy*

 Synoptic report for invasive malignant melanoma.

### 2.2. Summary Diagnosis

 Invasive malignant melanoma, Clark level 3, Breslow thickness 0.8 mm, closest side margin 1.25 mm, other side margin 2.5 mm, and closest deep margin 4.1 mm.
 Tumour type: invasive malignant melanoma arising in an area of melanoma in situ.
 Ulceration: nil.
 Tumour infiltrating lymphocytes: mild.
 Regression: nil.
 Lymphovascular invasion: nil.
 Perineural spread/neurotropism: nil.
 Mitotic rate: 0 per sq mm.
 Microscopic satellitosis: nil.
 Radial margin of excision: closest side margin 1.25 mm, other side margin 2.5 mm.
 Deep margin: closest deep margin 4.1 mm.
 Associated naevus: nil.
 Case also viewed by Dr. F.O. who agrees with the diagnosis; reported by Dr. H T., Anatomical Pathologist.
 Office data: nl/lm/as.
 Ordered by Sharad Paul.
 Observation date: 16 August 2014.

Histological report is detailed above, which reveals a nonulcerated tumour of 0.8 mm Breslow thickness, Clark level 3 invasive melanoma, arising in an area of melanoma in situ. A complete skin and lymph node examination revealed no other abnormalities. After reviewing the histopathology, this patient was managed with a wide local excision with 1 cm margins in keeping with standard guidelines for management of Stage 1A melanoma of skin.

## 3. Discussion

Dermatologists, surgeons, and skin cancer doctors are faced with an epidemic of skin cancer in Australia and New Zealand. Actinic keratoses and squamous cell carcinomas share multiple genomic mutations that suggest common origins [17]. It is well known that increases in p53 mutations are seen in sun-damaged skin, AK, and SCC [18]. Given the need to reduce unnecessary surgery as well as associated costs, researchers have turned their focus to topical applications to deal with skin cancer. Some prevailing topical treatments include 5-fluorouracil, diclofenac sodium, topical photodynamic therapy (PDT) with 5-aminolevulinic acid (ALA), and Imiquimod.

Given the clinical interest for TLR agonists in metastatic melanoma and indeed skin cancer, it is essential to determine the mechanism of action of Imidazoquinolines such as Imiquimod. Imiquimod has many cellular effects that stimulate Th-1 innate immunity. The drug's effects are mediated after binding to TLR 7, the receptor that is found on dendritic cells and monocytes. TLR 7 is also involved in regulation of cellular apoptosis. Following Imiquimod treatment, “immunologic memory” is established, and this differentiates this drug from other topical agents [19]. From Imiquimod's early use for genital warts, it was noted that a significant proportion of patients ended up being “non-responders.”

Some authors have been enthusiastic about the “field clearance” effects of Imiquimod—the concept of lymphatic transport of immune cells and factors with subsequent immunological curing of tumours, not only in the treated area, but also in “field” around the treatment site. Akkilic-Materna and colleagues suggest that their observations on the actions of Imiquimod support the concept of lymphatic transport of immune cells and factors with subsequent immunological curing of tumours, not only in the treated area, but also in the area between the Imiquimod application site and the regional lymph nodes—what they term the “lymphatic field clearance” [20]. Others have raised concerns about recurrence after Imiquimod use and whether Imiquimod may select more aggressive tumour cells or may just convey a natural course of tumour recurrence as we see with other treatment modalities [21].

Recurrence aside, there have been several reports of Imiquimod triggering keratoacanthomas and indeed infiltrating or aggressive SCC [22]. There has been also a report of a pulmonary embolism occurring after Imiquimod use [23]. The exact mechanism of inducing tumours remains unknown, although the exuberant immunological response is blamed—a sort of fighting “fire with fire” when utilizing immune-modulating agents that stimulate apoptosis.

There are now several reports that have supported the use of Imiquimod in amelanotic lentigo maligna [24], periocular lentigo maligna [25], facial lentigo maligna [26], and even large lentigo malignas prior to staged excision [27]. However given the reports of Imiquimod causing aggressive SCC, or, in our case, an invasive melanoma arising at the site of topical Imiquimod use, we would like to stress the importance of follow-up after Imiquimod use.

Schön and others have discussed that more pleiotropic antitumoural responses have to be considered when studying Imidazoquinolines. They demonstrated that Imiquimod is able to act not only as synthetic adjuvant but also as direct inducer of apoptosis for melanoma cells in vitro and in vivo. They concluded that cell death was exerted by apoptosis rather than necrosis and that this proapoptotic signal is selectively activated in melanoma cells, but* not* in primary human melanocytes [28].

Of course, in this case report, it is impossible to prove causal effect—other than to say that the melanoma arose at the exact Imiquimod treatment site. However, we believe it is prudent, given this case-study, to undertake ongoing surveillance of patients after Imiquimod use.

## References

[1] Harrison C. J., Jenski L., Voychehovski T., Bernstein D. I. (1988). Modification of immunological responses and clinical disease during topical R-837 treatment of genital HSV-2 infection. *Antiviral Research*.

[2] Gerster J. F., Lindstrom K. J., Miller R. L., Tomai M. A., Birmachu W., Bomersine S. N., Gibson S. J., Imbertson L. M., Jacobson J. R., Knafla R. T., Maye P. V., Nikolaides N., Oneyemi F. Y., Parkhurst G. J., Pecore S. E., Reiter M. J., Scribner L. S., Testerman T. L., Thompson N. J., Wagner T. L., Weeks C. E., Andre J.-D., Lagain D., Bastard Y., Lupu M. (2005). Synthesis and structure—activity-relationships of 1H-imidazo[4,5-c] quinolines that induce interferon production. *Journal of Medicinal Chemistry*.

[3] Salasche S. J., Levine N., Morrison L. (2002). Cycle therapy of actinic keratoses of the face and scalp with 5% topical imiquimod cream: an open-label trial. *Journal of the American Academy of Dermatology*.

[4] Beutner K. R., Geisse J. K., Helman D., Fox T. L., Ginkel A., Owens M. L. (1999). Therapeutic response of basal cell carcinoma to the immune response modifier imiquimod 5% cream. *Journal of the American Academy of Dermatology*.

[5] Smyth E. C., Flavin M., Pulitzer M. P. (2011). Treatment of locally recurrent mucosal melanoma with topical imiquimod. *Journal of Clinical Oncology*.

[6] Gagnon L. (2011). Imiquimod advantage. *Dermatology Times*.

[7] Stanley M. A. (2002). Imiquimod and the imidazoquinolones: mechanism of action and therapeutic potential. *Clinical and Experimental Dermatology*.

[8] Gibson S. J., Lindh J. M., Riter T. R., Gleason R. M., Rogers L. M., Fuller A. E., Oesterich J. L., Gorden K. B., Qiu X., McKane S. W., Noelle R. J., Miller R. L., Kedl R. M., Fitzgerald-Bocarsly P., Tomai M. A., Vasilakos J. P. (2002). Plasmacytoid dendritic cells produce cytokines and mature in response to the TLR7 agonists, imiquimod and resiquimod. *Cellular Immunology*.

[9] Kaisho T., Akira S. (2006). Toll-like receptor function and signaling. *Journal of Allergy and Clinical Immunology*.

[10] Tomai M. A., Imbertson L. M., Stanczak T. L., Tygrett L. T., Waldschmidt T. J. (2000). The immune response modifiers imiquimod and R-848 are potent activators of B lymphocytes. *Cellular Immunology*.

[11] Goh M. S. Y. (2006). Invasive squamous cell carcinoma after treatment of carcinoma in situ with 5% imiquimod cream. *Australasian Journal of Dermatology*.

[12] Campalani E., Holden C. A. (2013). Keratoacanthoma associated with the use of topical imiquimod. *Clinical and Experimental Dermatology*.

[13] Chapman M. S., Spencer S. K., Brennick J. B. (2003). Histologic resolution of melanoma in situ (lentigo maligna) with 5% imiquimod cream. *Archives of Dermatology*.

[14] Kamin A., Eigentler T. K., Radny P., Bauer J., Weide B., Garbe C. (2005). Imiquimod in the treatment of extensive recurrent lentigo maligna. *Journal of the American Academy of Dermatology*.

[15] Aspord C., Tramcourt L., Leloup C., Molens J. P., Leccia M. T., Charles J., Plumas J. (2014). Imiquimod inhibits melanoma development by promoting pDC cytotoxic functions and impeding tumor vascularization. *Journal of Investigative Dermatology*.

[16] Bong A. B., Bonnekoh B., Franke I., Schön M. P., Ulrich J., Gollnick H. (2002). Imiquimod, a topical immune response modifier, in the treatment of cutaneous metastases of malignant melanoma. *Dermatology (Basel, Switzerland)*.

[17] Ashton K. J., Weinstein S. R., Maguire D. J., Griffiths L. R. (2003). Chromosomal aberrations in squamous cell carcinoma and solar keratoses revealed by comparative genomic hybridization. *Archives of Dermatology*.

[18] Brash D. E., Ziegler A., Jonason A. S., Simon J. A., Kunala S., Leffell D. J. (1996). Sunlight and sunburn in human skin cancer: p53, apoptosis, and tumor promotion. *Journal of Investigative Dermatology Symposium Proceedings*.

[19] McGillis S. T., Fein H. (2004). Topical treatment strategies for non-melanoma skin cancer and precursor lesions. *Seminars in Cutaneous Medicine and Surgery*.

[20] Akkilic-Materna M., Massone C., Komericki P. (2011). Imiquimod and lymphatic field clearance: a new hypothesis based on a remote immune action on skin cancer. *Acta Dermato-Venereologica*.

[21] Skaria A. M. (2013). Facial basal cell carcinomas recurring after imiquimod therapy. *Dermatology*.

[22] Fernández-Vozmediano J., Armario-Hita J. (2005). Infiltrative squamous cell carcinoma on the scalp after treatment with 5% imiquimod cream. *Journal of the American Academy of Dermatology*.

[23] Leroy B., Wolf F., Descotes J., Vial T. (2012). Imiquimod and pulmonary embolism: a case report. *Fundamental and Clinical Pharmacology*.

[24] Lapresta A., García-Almagro D., Sejas A. G. (2012). Amelanotic lentigo maligna managed with topical imiquimod. *The Journal of Dermatology*.

[25] O'Neill J., Ayers D., Kenealy J. (2011). Periocular lentigo maligna treated with imiquimod. *Journal of Dermatological Treatment*.

[26] Ventura F., Rocha J., Fernandes J. C., Pardal F., Brito C. (2009). Topical imiquimod treatment of Lentigo Maligna. *Case Reports in Dermatology*.

[27] Cotter M. A., McKenna J. K., Bowen G. M. (2008). Treatment of lentigo maligna with imiquimod before staged excision. *Dermatologic Surgery*.

[28] Schön M., Bong A. B., Drewniok C., Herz J., Geilen C. C., Reifenberger J., Benninghoff B., Slade H. B., Gollnick H., Schön M. P. (2003). Tumor-selective induction of apoptosis and the small-molecule immune response modifier imiquimod. *Journal of the National Cancer Institute*.

